# Hypothesis: genetic and epigenetic risk factors interact to modulate vulnerability and resilience to FASD

**DOI:** 10.3389/fgene.2014.00261

**Published:** 2014-08-05

**Authors:** Elif Tunc-Ozcan, Laura J. Sittig, Kathryn M. Harper, Evan N. Graf, Eva E. Redei

**Affiliations:** Department of Psychiatry and Behavioral Sciences, Northwestern UniversityChicago, IL, USA

**Keywords:** prenatal ethanol, strain differences, allele specific expression, rat, thyroid hormones, second generation

## Abstract

Fetal alcohol spectrum disorder (FASD) presents a collection of symptoms representing physiological and behavioral phenotypes caused by maternal alcohol consumption. Symptom severity is modified by genetic differences in fetal susceptibility and resistance as well as maternal genetic factors such as maternal alcohol sensitivity. Animal models demonstrate that both maternal and paternal genetics contribute to the variation in the fetus' vulnerability to alcohol exposure. Maternal and paternal genetics define the variations in these phenotypes even without the effect of alcohol *in utero*, as most of these traits are polygenic, non-Mendelian, in their inheritance. In addition, the epigenetic alterations that instigate the alcohol induced neurodevelopmental deficits can interact with the polygenic inheritance of respective traits. Here, based on specific examples, we present the hypothesis that the principles of non-Mendelian inheritance, or “exceptions” to Mendelian genetics, can be the driving force behind the severity of the prenatal alcohol-exposed individual's symptomology. One such exception is when maternal alleles lead to an altered intrauterine hormonal environment and, therefore, produce variations in the long-term consequences on the development of the alcohol-exposed fetus. Another exception is when epigenetic regulation of allele-specific gene expression generates disequilibrium between the maternal vs. paternal genetic contributions, and thereby, modifies the effect of prenatal alcohol exposure on the fetus. We propose that these situations in which one parent has an exaggerated influence over the offspring's vulnerability to prenatal alcohol are major contributing mechanisms responsible for the variations in the symptomology of FASD in the exposed generation and beyond.

## Introduction

Alcohol consumption during pregnancy can result in fetal alcohol spectrum disorder (FASD), a constellation of disabilities including deficient pre- and postnatal growth, morphological malformations of the face and/or brain, and cognitive and behavioral deficits (Manning and Eugene Hoyme, 2007). These teratological outcomes vary significantly among individuals with respect to range and severity, even after allowing for the effects of timing, duration, and amount of alcohol exposure. This strongly suggests that genetic vulnerability may contribute to the etiology of FASD. We hypothesize that genetic sensitivity of the mother and the fetus to the direct and indirect effects of alcohol on epigenetically regulated genes leads to the individual variations observed in the severity of FASD symptoms.

It has been shown in mouse models that the same maternal alcohol exposure protocol in different inbred mouse strains results in a spectrum of affectedness from severe malformations to no teratogenesis, depending on the strain (Downing et al., 2009). Thus, vulnerability to fetal alcohol, as manifested in specific phenotypes, could be analyzed as a quantitative trait. Few studies have attempted this in mouse (Browman and Crabbe, 2000; Anthony et al., 2010; Chen et al., 2011; Downing et al., 2012), and we are unaware of any human studies trying to map loci for fetal alcohol vulnerability using a non-candidate gene approach. The difficulty lies in part in mapping disease genes for complex traits *per se*. In addition, the vulnerability to fetal alcohol can differ for each of the many different endophenotypes, ranging from gross morphological effects to subtle neurobehavioral changes, and these specific endophenotypes most likely have differing polygenic contributions. Another reason for the difficulty to identify genetic contribution to FASD is that genetic and epigenetic effects in FASD are highly interactive, defying simple associations (Rakyan et al., 2011; Liu et al., 2013). Therefore, animal models present important opportunities for discovering new candidate mechanisms and pathways toward the understanding of the etiology of FASD.

## Non-canonical maternal and paternal genetic contributions

### Programming of offspring's health by in utero environment: maternal genetic effects

We argue that fetal programming influenced by the *in utero* environment can interact with genetic sources of vulnerability. Fetal programming is “epigenetic” rather than genetic since it affects F1 phenotypes via *in utero* programming rather than by inherited DNA sequence *per se*. In a series of classic studies conducted in the early 1990s, Barker and his group observed the first “fetal origin of adult disease” phenomenon, whereby the prenatal environment influences the phenotype of adult offspring (Barker et al., 1989, 1990, 1993). The authors revealed a negative correlation between size at birth and future incidence of cardiovascular and metabolic disease, including hypertension (Barker et al., 1990), ischemic heart disease (Barker et al., 1989), and non-insulin dependent diabetes (Barker et al., 1993). The association between lowered fetal and infant weight and subsequent type 2 diabetes, hypertension, and hyperlipidemia was confirmed in two individual cohorts born during different time periods (Barker et al., 1993). They also hinted at the mechanisms, suggesting that in the face of poor early nutrition, the fetus undergoes endocrine and metabolic fetal adaptations.

**Figure 1:**
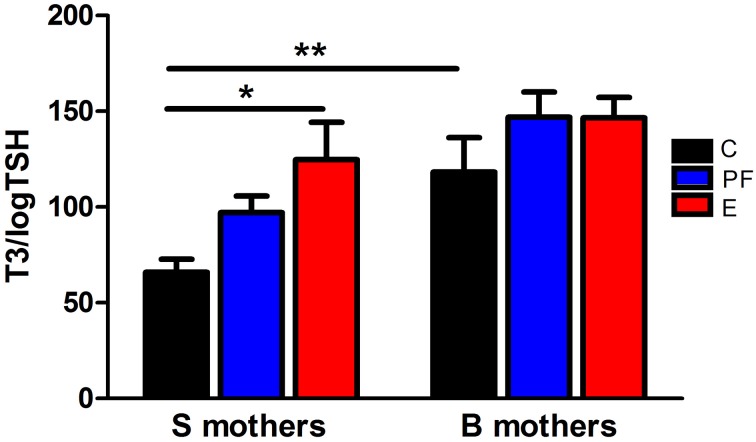
**Pregnant Sprague Dawley (S) and Brown Norway (B) dams differ in their thyroid function and thyroid hormonal response to alcohol**. Diet administration and prenatal diet groups are routinely used in our lab and are the same throughout the following experiments. Female S or B dams were divided into 3 prenatal treatment groups: C, control; PF, pair-fed; and E, ethanol. Control dams were kept on conventional laboratory chow *ad libitum* throughout their pregnancy. The PF and E treatment groups received liquid diet (Lieber-DeCarli'82; Bio-Serv. Frenchtown, NJ, USA) starting at gestational day (GD) 4. Ethanol diet began at GD8, and from GD8 to 10 the percentage of E in the diet was increased until it reached 5% (w/v) and then was kept constant until GD20. Each individual PF dam received a liquid diet that was isocaloric to the amount consumed by an individual E dam on the previous GD. On GD21, plasma levels of total T3 and TSH were measured by radioimmunoassay as previously described (Sittig and Redei, 2010). The T3/logTSH ratio was derived as a measure of thyroid function. The T3/logTSH ratios were lower in S mothers than B mothers [strain *F*_(1, 51)_ = 13.94, *p* < 0.01] and alcohol consumption significantly altered this ratio [diet *F*_(2, 51)_ = 5.04, *p* < 0.05]. Data were analyzed by Two-Way ANOVA; Bonferroni *post-hoc* test results are shown. Values are mean ± s.e.m. ^*^p < 0.05, ^**^p < 0.01, N = 9–11/group. This figure is modified from Sittig and Redei (2010).

**Figure 2:**
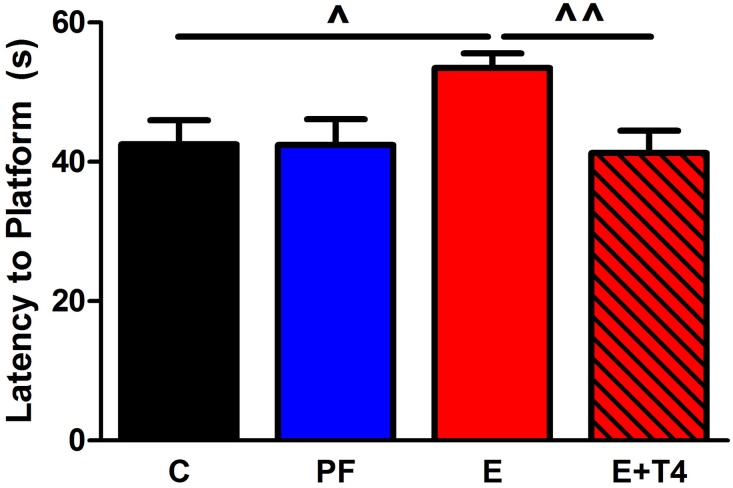
**Thyroxine (T4, 30 μg/ml) in the ethanol-containing liquid diet during gestation ameliorates spatial learning/memory deficits in adult offspring of Sprague Dawley (S) dams**. Adult male and female SS offspring (80–90 days old) of C, PF, E and ethanol+thyroxine (E+T4) were trained in the Morris Water Maze to locate a hidden platform. The E+T4 group received 30 μg/ml T4 (Sigma-Aldrich Co, St Louis, MO, USA) in the E-containing liquid diet, which, based on the daily diet consumption, is equivalent to approximately 3 mg/100 gBW/day of T4. Training consisted of four trials daily for six consecutive days. No sex differences were found, so male and female data were combined. In the last day of the test, E adult offspring still showed higher latency to reach the platform, which was reversed by maternal T4 supplementation [*F*_(3, 23)_ = 3.26, *p* < 0.05]. Data were analyzed by One-Way ANOVA; hypothesis testing is by Student *t*-test shown in the figure. Values are mean ± s.e.m. ^^^*p* < 0.05, ^^^^*p* < 0.01, *N* = 6/group. This figure is modified from Wilcoxon et al. (2005).

Fetal programing by alcohol includes changes in maternal and fetal hormone levels that exert long-lasting consequences. For example, increased or decreased levels of glucocorticoids of the pregnant dams with or without *in utero* alcohol exposure can affect the neuroendocrine stress-response of the progeny (Mcgivern and Redei, 1994; Slone and Redei, 2002; Wilcoxon et al., 2003; Glavas et al., 2007; Hellemans et al., 2010; Brunton and Russell, 2011; Liang et al., 2011). Our work illustrates that maternal thyroid hormones can superimpose additional phenotypic consequences to the underlying genetic susceptibilities to prenatal alcohol inherent in specific strains of rats (Sittig et al., 2011b). We have shown that Sprague Dawley (S) rat dams have lower plasma triiodothyronine (T3) and higher thyroid stimulating hormone (TSH) levels than Brown Norway (B) dams (Figure 1) (Sittig and Redei, 2010). Moreover, the thyroid function of S dams is more labile, as shown by their increased T3/logTSH ratio after alcohol consumption, in contrast to the stable measures in the B dams. S dams on alcohol-containing diet also show significantly lower plasma free T4 levels compared to those of B dams (Sittig and Redei, 2010). This maternal genetic susceptibility makes their fetus exceedingly vulnerable by lowering *in utero* free thyroxine (T4) levels, which are relevant and critical for fetal brain development. Supplementation of the vulnerable S dam with T4 during alcohol consumption ameliorates the memory deficits observed in adult offspring (Figure 2) (Wilcoxon et al., 2005), supporting this premise. Thus, decreased levels of maternal T4, reduced by alcohol consumption in combination with the alleles for the *a priori* lower thyroid hormones in the S mother, are risk factors for the developing fetus. Although this vulnerability is the consequence of maternal genetic differences, it is epigenetic in terms of the mechanism by which it ultimately affects the fetus.

The interaction between *in utero* environmental challenges and maternal genetic effects can result in increased vulnerability or relative resilience to these challenges, as illustrated above. Studies of genetic vulnerability to alcohol exposure in mice have cited maternal genetic effects as playing an important role in the vulnerability of offspring (Gilliam and Irtenkauf, 1990; Gilliam et al., 1997; Downing et al., 2009). Such effects were seen when progeny of reciprocal crosses of two differentially susceptible strains showed differences in the consequences of exposure to prenatal alcohol, based on the maternal strain (Gilliam and Irtenkauf, 1990). Although these offspring are genetically identical, their maternal strain has a strong influence on fetal vulnerability to the environmental (alcohol exposure) insult. Similarly, we found that offspring of the vulnerable alcohol-consuming S dams show social behavioral deficits, while the genetically identical offspring of B dams do not (Sittig et al., 2011b).

### Paternal genetic effects

**Figure 3:**
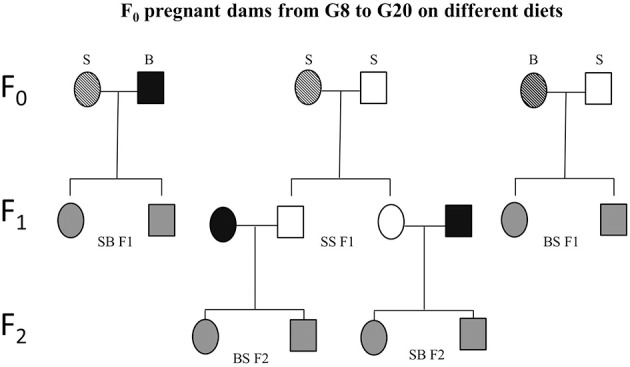
**Schematic experimental design**. Sprague Dawley (S) or Brown Norway (B) females (F_0_) were mated with either S or B males. From gestational day 8 (G8) through 20 (G20), these dams were exposed to one of four prenatal treatments C, PF, E, or E+0.3 μg/ml T4. Based on the daily diet consumption, dams receive the equivalent of 8 μg/100 gBW/day of T4 in the E+T4 group. The resulting SS, SB, and BS (maternal strain first) first generation (F1) offspring were employed in the different tests and/or mated. Experimentally naïve SS F1 offspring of all four treatment groups were mated with naïve male and female B mates. The dams used to generate the BS F2 and SB F2 offspring did not consume ethanol while pregnant. Circles designate females and squares designate males. Open fill are S, black fill are B rats, and gray fill indicate F1 or F2 crosses between B and S. A strip pattern indicates alcohol consumption during pregnancy.

The ability to experimentally observe the paternal influence requires two different paternal strains, and that the two strains used in the experiment be phenotypically different for the trait being studied. An important paradigm for studying maternal vs. paternal genetic effects is the reciprocal F1 design, using one strain of rat as the alcohol-consuming mother (S or B) and varying the strain used as the father, B or S, respectively (Figure 3). Evidence from this paradigm has shown the extent of paternal influence on fetal vulnerability. Specifically, BS F1 fetuses (maternal strain is first) are more vulnerable than BB fetuses to the alcohol-induced decrease in fetal body weight (Sittig and Redei, 2010). Although the specific genes/alleles underlying fetal body weight deficit vulnerability remain to be elucidated, the above experiment provides proof that this particular vulnerability is subject to paternal genetic influence. Moreover, it is worth noting that since the paternal strain significantly influences the body weight of offspring, a measure of vulnerability that was previously thought to depend mostly on how much the mother drinks and her investment of nutritional resources in the fetus, paternal genetic influence could be considerable in other FASD-related phenotypes as well.

**Figure 4:**
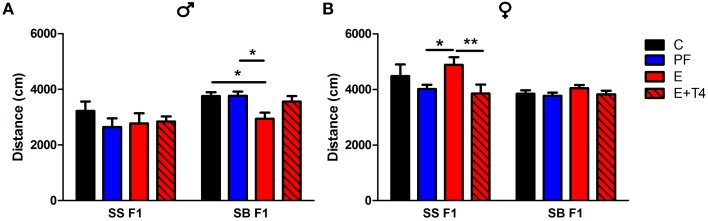
**Prenatal alcohol affects activity of adult offspring depending on paternal genetic background in the open field test**. Adult male **(A)** and female **(B)** rats of the SS and SB genetic backgrounds (see Figure 3) were exposed to four prenatal treatments (C, PF, E, or E+0.3 μg/ml T4) as described previously (Tunc-Ozcan et al., 2013). They were tested in a novel, 82 cm diameter circular open field arena with a light intensity of 60 lux for 10 min. Distance traveled in cm was analyzed from recordings by TSE videomot 2 software (version 5.75, Bad Homburg, Germany). In males, total distance traveled was decreased by *in utero* ethanol exposure only if the paternal genetic background is B [*F*_(1, 80)_ = 14.14, *p* < 0.01], while in females, increased activity with *in utero* ethanol exposure was found only with the paternal genetic background of S [*F*_(1, 79)_ = 9.38, *p* < 0.01]. Data were analyzed by Two-Way ANOVA, Bonferroni *post-hoc* results are shown. Values are mean ± s.e.m. ^*^p < 0.05, ^**^p < 0.01, *N* = 6–18/group.

Further effects of paternal genetic background can be seen in the differences in activity levels between SS and SB F1 males in the open field test (OFT). The activity levels, as measured by the OFT show that there is a paternal genetic effect. SB F1 males exposed to prenatal alcohol show hypoactivity in the OFT (as measured by distance moved), when compared to their genetically identical control, PF and T4 treated cohort. This decrease in activity is not observed in SS F1 cohort, as there are no treatment effects in this genetic background (Figure 4A). In contrast, SS F1 E females showed hyperactivity compared to their nutritional control, while SB F1 females did not show this phenotype (Figure 4B). Since the maternal *in utero* environment is the same for these two sets of F1s, their differential response to prenatal alcohol can be attributed to the vulnerability conferred by the paternal genetic influence. Interestingly, both of these alterations in activity induced by prenatal alcohol were reversed by administration of a low dose (0.3 μg/ml) T4 to the alcohol-consuming pregnant dam.

## Epigenetic vulnerability

Genomic imprinting is an epigenetic phenomenon that affects physiological and behavioral programming of the offspring. It is defined as the differential expression of the maternal and paternal alleles for particular gene(s) in certain genomic loci and thought to be controlled primarily by combinations of epigenetic modifications (i.e., DNA methylation, expression of various types of noncoding RNAs and histone tail modifications, such as acetylation, methylation etc.) (Delaval and Feil, 2004; Mazzio and Soliman, 2012; Seisenberger et al., 2013). The epigenetic marks that maintain the differential allelic expression can be sex-, developmental stage-, and tissue-specific. In addition to the placenta, the brain has been shown to exhibit enriched imprinting (Gregg et al., 2010). These paternal and maternal epigenetic “imprints” are created during gametogenesis and carried in sperm and oocytes, respectively. Immediately after conception, DNA methylation marks on the parental gametes are erased in two waves of de-methylation. First, the paternal pronucleus undergoes rapid de-methylation in the zygote followed by a passive loss of DNA methylation marks in the maternal genome. Subsequently a wave of global re-methylation occurs in the early embryo, whereby different cell lineages are re-methylated appropriately, but often differently. DNA methylation at the differentially methylated regions of imprinted genes are reset in primordial germ cells but are protected from reprogramming in the early embryo (Seisenberger et al., 2013; Skinner et al., 2013). The time frame of both de-methylation and re-methylation differs between male and female embryos, providing the possibility for sex-specific imprinting differences to occur (Reik et al., 2001; Seisenberger et al., 2013).

During re-methylation, the embryo is more sensitive to environmental perturbations that affect the methylation status at important regulatory loci (Feil and Fraga, 2011). For example, humans prenatally exposed to the Dutch “hunger winter” famine of 1944, showed decreased DNA methylation at the differentially methylated region associated with insulin-like growth factor 2 (*IGF2*). This altered methylation was detected approximately six decades after the original environmental insult (Heijmans et al., 2008), which directly illustrates the power of environmental insults to induce long-term, physiologically-relevant epigenetic changes. A growing body of literature implicates alcohol as a potent epigenetic modifier during prenatal development. Fetal alcohol exposure alters genomic imprinting at the *H19-Igf2* locus (Downing et al., 2011; Stouder et al., 2011; Knezovich and Ramsay, 2012) and at *Rasgrf1* (Knezovich and Ramsay, 2012). In addition, it is implicated in long-lasting alterations in DNA methylation in imprinted domains that harbor non-coding RNAs (Balaraman et al., 2013; Laufer et al., 2013). Together, these data support the “fetal origin of adult disease” hypothesis predicted by Barker (2004).

Here we will provide examples from our own work, which illustrate that small, brain-regional variations in complex gene expression patterns can influence the severity of outcome in an FASD model.

### Changes in allelic gene expression: brain region- and sex-specificity

**Figure 5:**
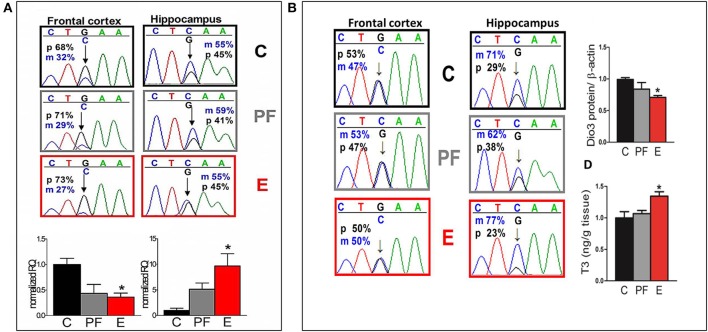
**(A)** Allele-specific expression of *Dio3* is brain region specific, preferentially paternal in the fetal frontal cortex and slightly maternal in the fetal hippocampus; prenatal alcohol inversely affects expression of total *Dio3* in these regions. Representative sequence traces of *Dio3* transcripts containing the SNP between Sprague Dawley (S) (“C”) and Brown Norway (B) (“G”) strains in male fetal SB frontal cortices, and hippocampi from three prenatal treatment groups (C, PF, and E). Pyrosequencing means of paternal (p; black = G) and maternal (m; blue = C) allelic *Dio3* ratio are inset onto individual fetal sequence traces. Total *Dio3* mRNA levels were measured by quantitative real-time RT-PCR in male fetal frontal cortices, and hippocampi from the three prenatal treatment groups. Relative quantification values are normalized to the appropriate control group. *N* = 3–7/prenatal treatment/brain region. **(B)** The effects of prenatal alcohol on adult *Dio3* imprinting is hippocampus specific and leads to functional consequences. Representative sequence traces of *Dio3* in adult SB frontal cortices and hippocampi derived from individual male offspring from the three prenatal treatment groups (C, PF, and E). Dio3 protein levels were measured by Western blot and normalized to β-actin levels in the adult SB male hippocampus. Free T3 was measured by radioimmunoassay after extraction from individual samples and the T3/wet tissue weight values (ng/g) were normalized to controls in the adult SB male hippocampus. *N* = 4–6/prenatal treatment/brain region. Data were analyzed by One-Way ANOVA, Bonferroni *post-hoc* results are shown. Values are mean ± s.e.m., ^*^p < 0.05 C vs. E. This figure is modified and shown by permission from Sittig et al. (2011b).

Since imprinted genes are epigenetically regulated, they are particularly vulnerable to disruptions induced by alcohol exposure during development (Haycock, 2009). Knowing this, we tracked maternal and paternal expression of the maternally imprinted gene that encodes the thyroid metabolizing enzyme deiodinase 3 (Dio3) in several brain regions of the SB F1 rat offspring (Sittig et al., 2011b). Our analysis shows a switch from primarily paternal expression in the fetal frontal cortex to slightly elevated maternal expression in the fetal hippocampus (Figure 5A) (Sittig et al., 2011a,b). Furthermore, transcript levels of *Dio3* are significantly influenced by maternal alcohol consumption, but in the opposite direction in these brain regions. Curiously, this brain region-specific difference in allelic expression of *Dio3* strengthens in adulthood (Figure 5B) (Sittig et al., 2011b). Specifically, while *Dio3* expression becomes mostly biallelic in the frontal cortex, it becomes clearly maternal in the hippocampus. Furthermore, fetal alcohol exposure exacerbates maternal-specific expression, thereby conferring a subsequent decrease in paternal contribution in the hippocampus. This allele-specific expression is accompanied by a corresponding decrease in *Dio3* enzyme levels and an increase in T3 levels. T3 is a substrate of *Dio3* (Figure 5B). These effects are sex-specific, occurring only in adult males, but not females (Sittig et al., 2011b). The patterns of changes in hippocampal thyroid hormone levels correspond to hippocampus-based deficits in social behavior and memory in males only (Sittig et al., 2011b).

Showing that relative allelic expression levels (maternal vs. paternal) of one gene can be tuned across brain regions leads to intriguing questions about the forces that shape differential evolution of gene expression control across brain regions. Should a certain brain region be favored by one parent or the other in control over expression of a specific imprinted gene, the consequences could include the biased inheritance of functions orchestrated by that brain region. This implies that parental bias can be transferred to the germ cells and that any epigenetic modification affecting the expressed allele could affect brain function of the progeny. If many genes are biased to maternal expression in the hippocampus, such as *Dio3*, then the hippocampus might be a site where maternal genetic influence trumps that of the father. In cases where the mother has known to have deleterious sequence variations, this could lead to predictions about hippocampus-specific functional deficits. In this way, these “exceptional” imprinted genes that give rise to highly tunable and therefore highly vulnerable gene expression patterns could be the ones that have the potential to influence vulnerability the most.

### Second generational effects of alcohol: maternal vs. paternal transmission

Evidence from both human and animal studies indicates that the second generation (F2) progeny incurs consequences of the first generation's exposure to alcohol (Rouleau et al., 2003; Kvigne et al., 2008; Govorko et al., 2012; Harper et al., 2014). These deficits could be transmitted to the next generation through a variety of mechanisms. Inheritance of the phenotype could occur indirectly, through alcohol-induced changes in hormonal programming of the F1 generation that affect their progeny (Govorko et al., 2012; Mead and Sarkar, 2014). In addition, prenatal alcohol exposure could permanently change the epigenetic landscape of the F2 gametes as they are developing in the F1 generation (Mead and Sarkar, 2014), conferring the phenotype on the F2 generation through their reprogrammed gametes. Animal models are proving exceptionally powerful for evaluating intergenerational effects since they allow complete control over environmental exposure. For example, we tested the intergenerational effects of maternal alcohol consumption using a rat model, where S pregnant dams received alcohol-containing liquid diet as described previously (Revskoy et al., 1997; Wilcoxon et al., 2005; Sittig and Redei, 2010), then their SS F1 offspring were allowed to grow to adulthood with no further alcohol exposure. Males and females from the F1 generation were mated to alcohol naive male and female Brown Norway (B) rats to generate matrilinear SB F2 and patrilinear BS F2 progeny (Figure 3). Adult offspring of all generations and crosses were tested in a glucose tolerance test (GTT).

**Figure 6:**
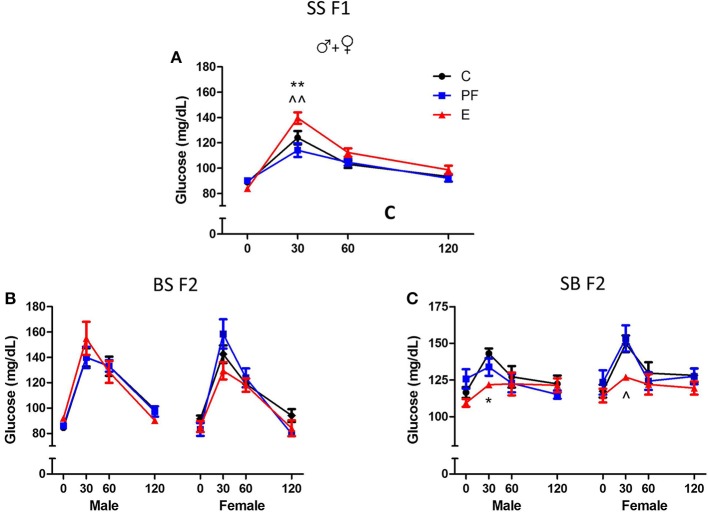
**Grandmaternal alcohol consumption during pregnancy affects serum glucose response to glucose tolerance test of grand-offspring via the maternal line**. **(A)** Adult Sprague Dawley (SS F1) male and female rats exposed to alcohol *in utero* showed hyperglycemic glucose responses in the glucose tolerance test (GTT). **(B)** Prenatal alcohol does not affect the BS F2 offspring of naïve B female mated to SS F1 males. **(C)** Male and female SB F2 progeny of SS F1 females exposed to alcohol prenatally, mated to naïve B males, are hypoglycemic. Animals were fasted overnight and blood was collected in the morning before, and 30, 60, and 120 min after an intraperitoneal injection of 2 g/kg body weight dextrose as described previously (Harper et al., 2014). Glucose levels were measured in duplicates by Stanbio glucose liquicolor kit. There were no sex differences in the glucose response to GTT of SS F1 offspring; therefore, male and female data were combined. Statistical analyses were conducted by appropriate ANOVA followed by Bonferonni *post-hoc* tests. *p* < 0.05 ^*^C vs. E, ^^^PF vs. E, ^+^C vs. PF. Data are presented as mean ± s.e.m.; *N* = 4–11/group. This figure includes data from Harper et al. (2014).

**Figure 7:**
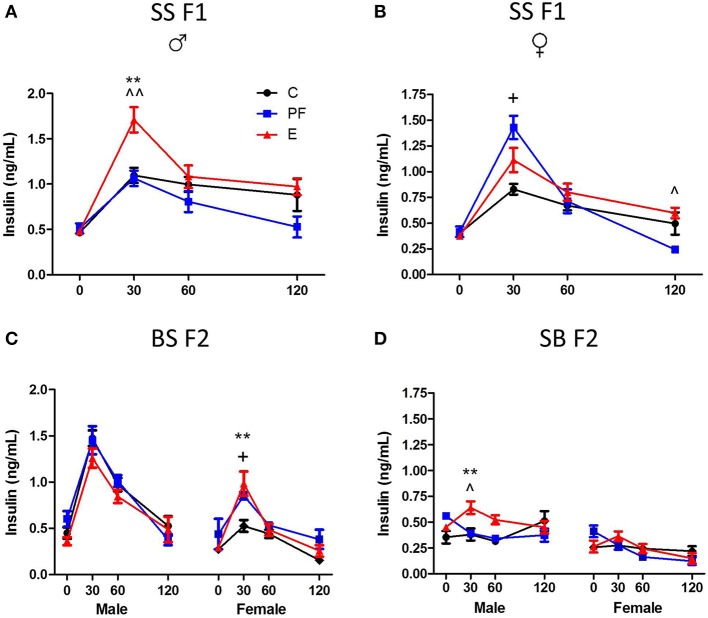
**Grandmaternal alcohol consumption during pregnancy affects serum insulin response to glucose tolerance test of grand-offspring via the maternal line**. **(A)** Prenatally alcohol exposed adult SS F1 males have dramatically greater insulin response in the GTT. **(B)** Female SS F1 offspring of dams on pairfed diet, but not on alcohol, are hyperinsulinemic. **(C)** Male BS F2 progeny of grandmaternal treatment groups show no differences in insulin response, while both PF and alcohol diet of grandmothers during gestation leads to hyperinsulinemis GTT profile of females. **(D)** SB F2 male progeny of SS F1 females exposed to alcohol prenatally is also hyperinsulinemic. GTT protocol is as described in Figure 6 and in Harper et al. (2014). Insulin levels were measured in duplicates by Ultra sensitive rat insulin ELISA kit. Statistical analyses were conducted by appropriate ANOVA followed by Bonferonni *post-hoc* tests. *p* < 0.05 ^*^C vs. E, ^^^PF vs. E, ^+^C vs. PF; ^**,^^^*p* < 0.01. Data are presented as mean ± s.e.m.; *N* = 4–11/group. This figure includes data from Harper et al. (2014).

Dams consuming E during pregnancy were hyperglycemic and their F1 offspring showed insulin resistance (Harper et al., 2014). Both males and females exposed to alcohol prenatally had hyperglycemic and hyperinsulinemic responses to GTT (Figures 6A, 7A,B). However, F2 progeny's responses to GTT varied depending on the sex of the prenatal alcohol exposed parent. Female offspring of males exposed to alcohol or PF diet prenatally showed hyperinsulinemic responses to GTT (Figure 7C). As the E did not differ from the PF effect, there was no patrilinear effect of grandmaternal alcohol exposure during pregnancy. In contrast, both male and female SB F2 progeny whose *mother* was exposed to ethanol *in utero* displayed hypoglycemic GTT response patterns (Figure 6C). Furthermore, a sex difference was seen in their insulin responses to GTT: male offspring presented hyperinsulinemic responses even though both male and female SB F2 progeny showed a flattened insulin and glucose response to GTT. Therefore, prenatal alcohol-induced dysregulation of glucose metabolism affected the matrilinear next generation because of the SS F1 female offspring's impaired glucose tolerance, which can put their progeny at risk for developing their own metabolic problems due to their intrauterine environment (Eberle and Ament, 2012).

Alternatively, primordial germ cells of the F1 offspring, while *in utero*, undergo alcohol-induced epigenetic changes in genes or genomic loci that contribute to abnormalities of glucose regulation in the F2 generation. Since the BS F2 progeny of SS F1 males do not show specific dysregulation of the GTT responses (Figures 6B, 7C), we can conclude that epigenetic changes leading to deficits in the F2 generation seem to be specific to the female F1 fetus. The mechanism of this vulnerability is not known, but is likely to include DNA methylation, since prenatal alcohol exposure induces changes in DNA methylation, and subsequently imprinting (Garro et al., 1991; Kaminen-Ahola et al., 2010; Downing et al., 2011). Future work is currently aimed at determining the epigenetic modifications responsible for the second generational effects of prenatal alcohol exposure, and whether they will transfer to the next, F3 generation.

## Genetic × epigenetic vulnerability

We presented evidence that highly individual and brain region-specific variability in allelic gene expression contributes significantly to the variable consequences of prenatal alcohol exposure observed in an FASD model. We illustrated above an additional layer of complexity, whereby genotype effects the epigenetic regulation of gene expression and the intergenerational transfer of the alcohol-induced endophenotype. These include not only the hippocampal strain-dependent and allele-specific changes in *Dio3* expression after prenatal alcohol exposure in the SB vs. BS F1 offspring, but also the altered glucose metabolism of SB vs. BS F2 progeny. One common denominator is that the SB cross results in a vulnerable offspring, while the BS cross seems to remain resistant to the maternal or grandmaternal effects of alcohol. For the intergenerational transfer of prenatal alcohol-induced deficit, the genetic × epigenetic vulnerability is complicated further by lineage effect due to prenatal alcohol exposure of the mother or the father. These data represents the first example of a genetic susceptibility and resilience based on parent-of-origin effects. It also shows a biological substrate for enhanced vulnerability to specific endophenotypes of FASD present in certain individuals.

### Potential genetic basis for genetic × epigenetic interactions

**Figure 8:**
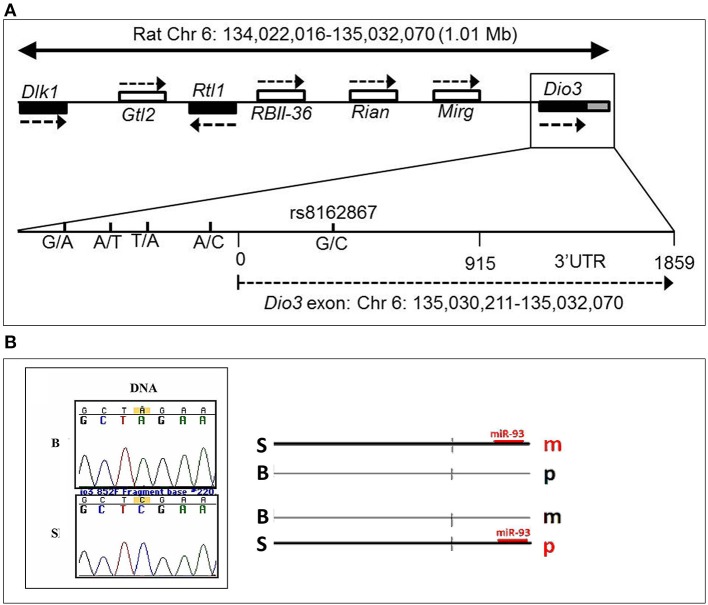
**(A)** Genomic location of the rat *Dlk1-Dio3* imprinted locus and the *Dio3* gene. Relative position of the paternally expressed genes *Dlk1, Rtl1*, and *Dio3* (filled boxes), and maternally expressed non-coding transcripts *Gtl2*, RBII-36 C/D snoRNAs, *Rian* and *Mirg* (open boxes). *Dio3* is located at the distal end of the imprinted locus and usually exhibits preferential paternal expression. *Dio3* contains a single exon (black) and 3′ untranslated region (gray). Specific subregion that was sequenced for polymorphisms between Brown Norway vs. Sprague Dawley and Long-Evans strains. Four polymorphisms were found within the high-GC promoter region (−500 to 0) proximal to the *Dio3* start site (0). A synonymous G/C SNP in the *Dio3* exon (342) allows determination of paternal/maternal allelic expression. Chromosomal bp location of the *Dio3* transcript is given below. Location of genes and non-coding transcripts are not to scale. This figure is shown by permission from Sittig and Redei (2012). **(B)** A Brown Norway SNP in the *Dio3* 3′UTR abolishes a putative miR-93 binding site. We have previously identified a C/A SNP between Sprague Dawley (S) and Brown Norway (B) strains in the *Dio3* 3′UTR (Dr. Laura Herzing, unpublished data). MicroInspector miRNA binding prediction program predicted a miR-93 binding site within the S sequence of the 3′UTR of *Dio3*, but not in the B sequence. This suggests that regulation by miR-93 binding to the S 3′UTR is only possible on the S allele. A schematic hypothetical illustration of how miR-93 binding to S but not B alleles could stabilize parent-of-origin *Dio3* alleles (m, maternal; p, paternal) to result in preferentially maternal expression in SB and preferentially paternal expression in BS hippocampus. This figure is shown by permission from Sittig (2012).

**Figure 9:**
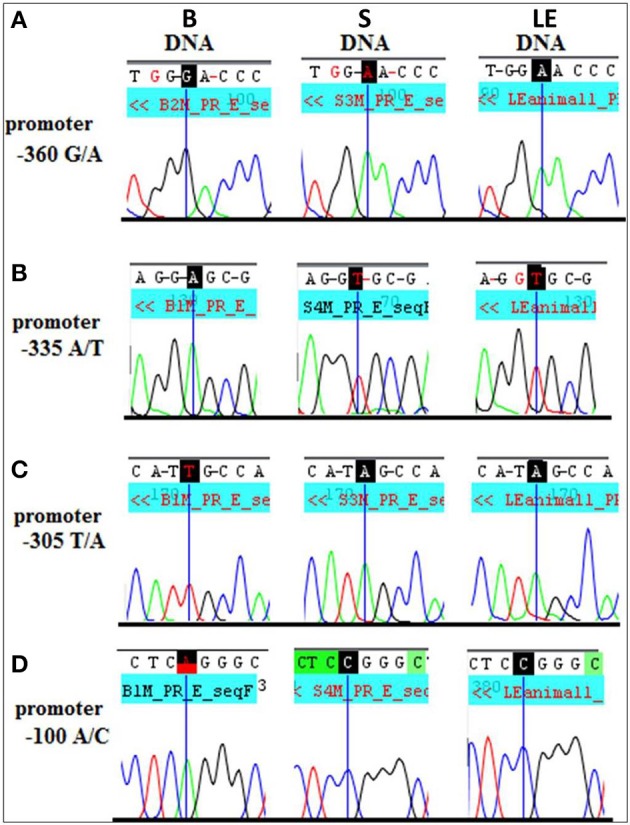
**Long Evans (LE) strain recapitulates the Sprague Dawley (S) genotype in the *Dio3* exon and its promoter**. Standard sequencing traces of four novel polymorphisms within the *Dio3* promoter region and their location relative to the *Dio3* start site are shown. **(A)** G/A at −360 bp upstream of *Dio3*; **(B)** A/T at −335 bp; **(C)** T/A at −305 bp; and **(D)** A/C at −100 bp. Brown Norway (B) genotype is given first, followed by S and LE. B genotype differs from the matching S and LE genotype at each polymorphic site. This figure is shown by permission from Sittig and Redei (2012).

What could be the mechanistic basis for such complex patterns of vulnerability to alcohol exposure? We hypothesized that the preferentially maternal expression of *Dio3* in the hippocampus of SB F1 animals vs. the preferentially paternal expression in the BS F1s (Sittig et al., 2011a) is due to sequence variations between the S and B strains at *Dio3* regulatory regions. Thus, alcohol may exaggerate this effect via epigenetic changes resulting in the differential effect of alcohol on hippocampal allelic *Dio3* expression in the SB vs. BS F1 offspring (Sittig et al., 2011b). Since S is the maternal strain in SB, but the paternal strain in BS, parent-specific hippocampal epigenetic marks could be affected by alcohol differently in these reciprocal crosses. To test the sequence variation hypothesis, we first mapped the hitherto unmapped *Dlk1-Dio3* imprinted region in the rat (Figure 8A) (Sittig and Redei, 2012). We identified four novel polymorphisms in the *Dio3* promoter region between these strains (Figure 8A). Furthermore, F1 offspring generated with another rat strain sharing these polymorphisms with S (Figure 9) have the same pattern of exaggerated maternal contribution to hippocampal *Dio3* expression (Sittig and Redei, 2012). Thus, any or all of these polymorphisms within the *Dio3* promoter could contribute to the differential effects of alcohol on SB vs. BS offspring.

In addition to the sequence variation between the S and B strains used for the allele-specific pyrosequencing of *Dio3*, we identified a second C/A SNP in the 3′ untranslated region (UTR) (Figure 8B). The 3′ UTR of *Dio3* may bind miRNA differently based on this sequence variation leading to differential allelic expression. To determine whether this SNP could affect the binding of target miRNAs to the 3′UTR of *Dio3*, we input both the S and B alleles as sequences into MicroInspector miRNA binding prediction program. Interestingly, the S allele permitted a binding site for miR-93 whereas the B SNP abolished the putative site. Expression data from microRNA.org indicated that rno-miR-93 (rat miR-93) is expressed, among other tissues, in the embryonic and adult hippocampus. A schematic indicating how miR-93 binding could stabilize the *Dio3* maternal allele for S animals and the paternal allele for B animals in the hippocampus is shown in Figure 8B. Positively correlated miRNA-mRNA interactions have been reported previously in the brain (Nunez et al., 2013), and should a similar mechanism be at play in the regulation of *Dio3* allelic expression in the hippocampus, the maternal S allele of SB progeny would be induced over its paternal B allele.

These are some of the many possibilities that may explain the individual susceptibility to FASD that is described and observed in animal models. We argue that there are many more instances where complex, brain region-specific, epigenetically regulated changes in gene expression underlie the manifestation of endophenotypes in FASD on an individual basis. By using animal models to demonstrate individual examples of complex vulnerability, we not only gain a conceptual understanding, but we build a mechanistic understanding of FASD that can be mined for major patterns, alleles, and biological pathways.

### What is next?

One could imagine that a few pathways may emerge as common “hits” where epigenetic and genetic vulnerabilities converge with specific functional consequences that correspond to common FASD pathophysiology (i.e., thyroid hormone homeostasis in brain). Although there is no limit on potential human (or animal) genetic variation, the development of a useful panel of assays that look at pathways affecting multiple FASD endophenotypes is possible. For example, the data on thyroid hormone related vulnerability to FASD that we have presented here show specific examples within a literature where altered thyroid hormone levels are implicated in placenta, brain, maternal and fetal blood in both rodents and humans exposed to alcohol (Heinz et al., 1996; Cudd et al., 2002; Wilcoxon and Redei, 2004; Liappas et al., 2006; Sittig and Redei, 2010; Shukla et al., 2011). We have begun to illuminate the “why” and “how” of thyroid hormone involvement in FASD by examining the genetic basis of thyroid hormone homeostasis, such as the imprinted domain containing *Dio3*. The answers turn out not to be simple, but they lead to specific targets for treatment and diagnosis. For example, alterations in thyroid hormone related markers in placenta are a promising source of functional readout for alcohol exposure at birth (Shukla et al., 2011). Furthermore, administration of low dose T4 to the alcohol-consuming dam can reverse the social interaction and spatial memory deficits in the adult offspring (Tunc-Ozcan et al., 2013).

## Conclusion

We argue for our hypothesis that aspects of genetic regulation that are considered exceptions to Mendelian genetics play an especially important role in FASD vulnerability. We point to hormonal changes in the maternal *in utero* environment, and parent of origin allelic gene expression differences as mechanisms that impact the first generation with direct intrauterine alcohol exposure, and which can potentially affect the second generation. Both mechanisms are based on non-Mendelian evolutionary systems that allow the parents to shape the offspring in preparation for the environment. We illustrate the complexity of such mechanisms by focusing on examples of alcohol-induced changes in the F0, F1, and F2 generations in a rat model of FASD. Specifically, we show brain region-specific changes as a result of prenatal alcohol exposure in the expression of an imprinted gene, which changes differ by maternal and paternal genotypes. Additional examples illustrate that maternal genetic vulnerability to alcohol can affect both F1 and F2 generations via altered maternal hormone levels and the subsequent *in utero* hormonal re-programming of the offspring. Both of these types of effects result in differences in vulnerability or resilience of the individuals to prenatal alcohol effects. Finally, we argue that these complex influences probably converge on final common pathways of which thyroid hormone homeostasis is an example, where known epigenetic and genetic vulnerabilities could be evaluated to improve clinical intervention.

## Author contributions

Experiments conceived and designed: Eva E. Redei and Laura J. Sittig. Performed: Laura J. Sittig, Elif Tunc-Ozcan, Kathryn M. Harper, and Evan N. Graf. Analyzed data: Eva E. Redei, Elif Tunc-Ozcan, Laura J. Sittig, Kathryn M. Harper, and Evan N. Graf. Wrote the manuscript: Laura J. Sittig, Eva E. Redei, and Elif Tunc-Ozcan. Edited and revised manuscript: Laura J. Sittig, Elif Tunc-Ozcan, Kathryn M. Harper, Evan N. Graf, and Eva E. Redei. Approved final version of manuscript: Laura J. Sittig, Elif Tunc-Ozcan, Kathryn M. Harper, Evan N. Graf, and Eva E. Redei.

### Conflict of interest statement

The authors declare that the research was conducted in the absence of any commercial or financial relationships that could be construed as a potential conflict of interest.
